# Development of a SARS-CoV-2 Vaccine Candidate Using Plant-Based Manufacturing and a Tobacco Mosaic Virus-Like Nano-Particle

**DOI:** 10.3390/vaccines9111347

**Published:** 2021-11-17

**Authors:** Joshua M. Royal, Carrie A. Simpson, Alison A. McCormick, Amanda Phillips, Steve Hume, Josh Morton, John Shepherd, Youngjun Oh, Kelsi Swope, Jennifer L. DeBeauchamp, Richard J. Webby, Robert W. Cross, Viktoriya Borisevich, Thomas W. Geisbert, Jennifer K. Demarco, Barry Bratcher, Hugh Haydon, Gregory P. Pogue

**Affiliations:** 1 Kentucky BioProcessing, Inc., Owensboro, KY 42301, USA; simpsoc3@kentuckybioprocessing.com (C.A.S.); phillia2@kentuckybioprocessing.com (A.P.); humes@kentuckybioprocessing.com (S.H.); mortonj2@kentuckybioprocessing.com (J.M.); shephej@kentuckybioprocessing.com (J.S.); ohy@kentuckybioprocessing.com (Y.O.); swopek@kentuckybioprocessing.com (K.S.); bratchb1@rjrt.com (B.B.); haydonh@kentuckybioprocessing.com (H.H.); pogueg1@RJRT.com (G.P.P.); 2 Department of Biological & Pharmaceutical Sciences, Touro University California, Vallejo, CA 95688, USA; amccormi@touro.edu; 3 Department of Infectious Disease, St. Jude Children’s Hospital, Memphis, TN 38105, USA; Jennifer.DeBeauchamp@stjude.org (J.L.D.); richard.webby@stjude.org (R.J.W.); 4 Galveston National Laboratory, Department of Microbiology and Immunology, University of Texas Medical Branch, Galveston, TX 77550, USA; rwcross@UTMB.EDU (R.W.C.); viborise@utmb.edu (V.B.); twgeisbe@utmb.edu (T.W.G.); 5 Center for Predictive Medicine for Biodefense and Emerging Infectious Diseases, School of Medicine, University of Louisville, Louisville, KY 40202, USA; jennifer.wolf.2@louisville.edu; 6 IC² Institute, University of Texas at Austin, Austin, TX 78805, USA

**Keywords:** SARS-CoV-2 vaccine, nanoparticle, receptor-binding domain, plant-based manufacturing

## Abstract

Stable, effective, easy-to-manufacture vaccines are critical to stopping the COVID-19 pandemic resulting from the coronavirus SARS-CoV-2. We constructed a vaccine candidate CoV-RBD121-NP, which is comprised of the SARS-CoV-2 receptor-binding domain (RBD) of the spike glycoprotein (S) fused to a human IgG1 Fc domain (CoV-RBD121) and conjugated to a modified tobacco mosaic virus (TMV) nanoparticle. In vitro, CoV-RBD121 bound to the host virus receptor ACE2 and to the monoclonal antibody CR3022, a neutralizing antibody that blocks S binding to ACE2. The CoV-RBD121-NP vaccine candidate retained key SARS-CoV-2 spike protein epitopes, had consistent manufacturing release properties of safety, identity, and strength, and displayed stable potency when stored for 12 months at 2–8 °C or 22–28 °C. Immunogenicity studies revealed strong antibody responses in C57BL/6 mice with non-adjuvanted or adjuvanted (7909 CpG) formulations. The non-adjuvanted vaccine induced a balanced Th1/Th2 response and antibodies that recognized both the S1 domain and full S protein from SARS2-CoV-2, whereas the adjuvanted vaccine induced a Th1-biased response. Both adjuvanted and non-adjuvanted vaccines induced virus neutralizing titers as measured by three different assays. Collectively, these data showed the production of a stable candidate vaccine for COVID-19 through the association of the SARS-CoV-2 RBD with the TMV-like nanoparticle.

## 1. Introduction

To date, seven coronaviruses (CoVs) capable of infecting humans have been identified, including severe acute respiratory syndrome CoV (SARS-CoV), Middle East respiratory syndrome CoV (MERS-CoV), and the newly identified CoV, SARS-CoV-2 [1]. Infection with any of these three viruses is associated with high morbidity and fatality rates [2]. SARS-CoV-2 has spread to >180 countries since December 2019. As of 13 June 2021, this virus has infected >175 million people and has been attributed to >3.5 million deaths worldwide [3]. The COVID-19 pandemic created worldwide health and economic crises. Although the number of new daily reported cases has been declining since May 2021, daily global deaths attributed to COVID-19 continue to oscillate. The emergence of new variants presents ongoing challenges to controlling the pandemic and an urgent need for the development of new vaccine platforms with the ability to adapt to new viral strains as they develop.

Vaccination is a proven strategy to reduce infection and attendant disease associated with communicable diseases [4]. Although several vaccines are now available, their availability remains limiting and additional platforms for rapid and cost-effective vaccine development would benefit society. Many vaccine platforms have been developed, including live-attenuated pathogens, inactivated pathogens, pseudotyped-virus vectors, nucleic acid-based vaccines, and protein subunit vaccines. The global pandemic caused by SARS-CoV-2 has stimulated vaccine development, including the development of manufacturing platforms [5]. Each approach offers different advantages but also suffers from particular limitations. Beyond the ability to stimulate robust humoral and cellular immunity, important considerations for a globally useful vaccine include the speed of manufacturing scale-up to a durable and consistent supply, vaccine stability, cold-chain requirements, and a simple delivery formulation.

Nanoparticle (NP)-based vaccines are an appealing platform. They can be provided through various routes of administration, stimulate potent innate and adaptive immune responses, and be easily tailored to address particular diseases or emerging viral variants [6,7]. Virus-like particles (VLP) are a type of NP, and VLP-based vaccines have previously proven to be clinically efficacious for hepatitis B virus and human papillomavirus [8]. Furthermore, NP presentation of antigens has been shown to enhance immunogenicity in animal models of respiratory syncytial virus, influenza, and various microbial diseases [9,10,11]. Plants have a long history as a manufacturing platform for VLPs and many plant-manufactured VLPs show promise in preclinical testing [12,13]. Indeed, a plant-produced quadrivalent VLP influenza virus vaccine completed Phase III clinical testing. When compared to a marketed quadrivalent vaccine, this plant-produced quadrivalent VLP induced hemagglutinin-inhibition titers and exhibited a similar efficacy in elderly populations and prevented influenza-related illness in test subjects [14].

Plant viruses are effective immunogens and have been used to study the function and maturation of antigen-presenting cells (APCs) in lymphoid tissues [15,16]. Plant viruses, and tobacco mosaic virus (TMV) in particular, have been used to present linear peptide epitopes and small immunological domains through conjugation or genetic fusion with their capsid proteins [15,17,18]. Although promising results have been observed in many preclinical systems, manufacturing challenges, such as recombinant virus solubility and poor recovery, and constraints associated with the genetic fusions, such as poor virus fitness, have limited this approach to a few immunogens.

We developed a VLP-type NP vaccine platform composed of recombinant vaccine antigens physically associated with the TMV virions [15]. This platform overcomes the limitations associated with TMV genetic fusions by conjugating an intact antigen to the assembled viral particle to maintain stimulatory activities; genetic fusions only tolerate small peptide fusions. Such TMV-based vaccines show robust uptake by and activation of dendritic cells in vitro [13,16,17]. The TMV antigen-presentation system is flexible and has been used to produce vaccines for five viral pathogens, three influenza, and two papillomaviruses, and two bacterial pathogens, *Yersinia pestis* and *Francisella tularensis* [18,19,20,21,22,23]. Several have efficacy in preclinical studies [19,20,22]. Furthermore, a quadrivalent influenza vaccine comprised of an equal mass mixture of four distinct TMV-hemagglutinin conjugates representing each of the 2020 viral strains is currently in Phase I clinical evaluation (NCT04439695).

Here, we described a TMV-based NP vaccine candidate that displays the SARS-CoV-2 receptor-binding domain (RBD) from the virus spike glycoprotein (S). This domain is required for human host cell receptor, ACE-2, binding and is a target for neutralizing antibodies [24]. We showed that the manufacturing process for the vaccine CoV-RBD121-NP is robust and that the vaccine is stable at 2–8 °C or 22–28 °C The CoV-RBD121-NP candidate vaccine induced high neutralizing titers in mice as measured by three different methods in vitro.

## 2. Materials and Methods

### 2.1. Vaccine Construction

The SARS-CoV-2 RBD antigen incorporated into CoV-RBD121-NP was derived from amino acids 331–632 from the Wuhan-Hu-1 novel coronavirus strain (GenBank ID: MN9087947.3) and fused to a human IgG1 Fc domain (171 allotype; GenBank ID: AAA02914.1). The *Nicotiana benthamiana* (Nb) extensin signal peptide was genetically fused to the N-terminus of the chimeric antigen to facilitate secretion and folding in the endomembrane system of the plants. Briefly, antigen expression and purification were performed through transient transfection of wild-type Nb plants with the expression vector (pKBP121) inducing the production of the antigen CoV-RBD121. Transfected plants were harvested, soluble protein fractions were isolated, and CoV-RBD121 was purified through a combination of Protein-A affinity and anion exchange chromatography. A modified TMV NP with an N-terminal lysine mutation (TMV NtK) was produced by infection of wild-type Nb plants with TMV NtK virions [18]. Infected tissue was harvested, the soluble protein was isolated, and virions were purified through a combination of Capto-Q and Capto-Core 700 chromatography. TMV NtK was inactivated and sterilized by exposure of the virus to ultraviolet light at 254 nm (UV_254_) at 5142 J/m^2^ within an ISO 5 environment. Inactivation was confirmed by virus infectivity assays.

Subsequently, TMV NtK was chemically conjugated to CoV-RBD121. Prior to conjugation, CoV-RBD121 was diafiltered into an MES-buffered salt solution and filtered through a 0.2 µm filter. Prior to conjugation, UV-inactivated TMV NtK was diafiltered into 5 mM sodium acetate buffer. The conjugation reaction was performed in a sterile, single-use closed system with bio-weld and aseptic linkages connecting all vessels, pumps, and filtration units using silicone and thermoplastic elastomer materials. Antigen was conjugated to the purified TMV NtK using EDC (1-ethyl-3-(3-dimethylaminopropyl)carbodiimide hydrochloride) and Sulfo-NHS (N-hydroxysulfosuccinimide) chemistries in a 1-h mixing reaction. The unreacted esters were quenched by the addition of an amine (Tris-buffer). Residual EDC, Sulfo-NHS, and Tris were removed by 10× diafiltration using a 30 kDa ultrafiltration membrane. The conjugated drug substance was diafiltered and formulated in a phosphate-buffered solution with 0.01% thimerosal as a preservative. Certificate analysis was performed to ensure product concentration, potency, purity, polydispersity, pH, lack of endotoxin, and appearance.

### 2.2. Transmission Electron Microscopy

Transmission electron microscopy (TEM) was conducted by NanoImaging Inc. (San Diego, CA, USA). Briefly, samples were diluted to working concentrations of ~0.05 mg/mL using a supplied final fill buffer. Each sample was imaged over a layer of continuous carbon supported by nitrocellulose on a 400-mesh copper grid. Grids were prepared by adding 3 µL of sample, blotting away excess with filter paper, and immediately staining with uranyl formate. TEM was performed using an FEI Tecnai T12 electron microscope operating at 120 keV equipped with an FEI Eagle 4k × 4k CCD camera.

### 2.3. Analytical Ultracentrifugation

TMV NtK and conjugate vaccines were diluted to the target concentration and loaded into cells with 2-channel charcoal-epon centerpieces with 12 mm optical pathlength. Loaded cells were placed in an AN-50Ti analytical rotor and separated at 9000 rpm in a Beckman-Coulter ProteomeLab XL-A analytical ultracentrifuge. Scans were recorded every 4 min for 4 h (60 scans per sample). Data were analyzed by Schuck 2000 using the SEDFIT (vs. 11.3) software (National Institutes of Health, Bethesda, MD, USA). The f/fo values were varied to find the best overall fit for the data for each sample. A second derivative regularization probability of 0.683 was used, and time-invariant noise was removed. The resultant size distributions were graphed, and peaks were integrated using OriginLab Origin vs. 9.0.0 (OriginLab, Northampton, MA, USA).

### 2.4. In Vitro Binding Assays

In vitro binding to CR3022 antibody and ACE2 was performed with the VaxArray Coronavirus Spike Protein Kit (VXCV-9000) and VaxArray Imaging System (InDevR, Boulder, CO, USA) according to the manufacturer’s instructions. Briefly, samples of CoV-RBD121 or CoV-RBD121-NP were lysed in Zwittergent 3-14 (EMD Millipore) in Protein Blocking Buffer (PBB, VX-6305) for 30 min at room temperature and then diluted in PBB and applied to a microarray capable of detecting binding to both CR3022 antibody and ACE2 and incubated in the supplied humidity chamber (VX-6200) for 60 min. After incubation, solutions were removed from the array using a multichannel pipet. Anti-Coronavirus Spike Protein Detection Label (VXCV-7630) was then applied to each well and the microarray slide was incubated in the humidity chamber for 30 min. The microarrays are then washed once with diluted Wash Buffers 1 (VX-6303) and 2 (VX-6304), 70% ethanol, and ultrapure water. Fluorescence was detected with the VaxArray Imaging System, and values were transferred to GraphPad (San Diego, CA, USA) for calculation of EC_50_ values.

### 2.5. Animal Immunization and Testing

Groups of five C57BL/6 female mice (7–8 weeks of age)/group were immunized by subcutaneous (s.c.) dosing once at day 1 and day 14 of study, with either 15 µg or 45 µg vaccine with or without 0.05 mg CpG (isotype 7909; Nitto Denko Avecia, Inc., Cincinnati, OH, USA) in 50 µL total volume. Controls included PBS (vehicle) and 45 µg of CoV-RBD121 alone (antigen not conjugated to TMV NtK). Treatment groups were as follows: PBS, CoV-RBD121 45 µg, CoV-RBD121-NP 15 µg, CoV-RBD121-NP 15 µg + CpG, CoV-RBD121-NP 45 µg, and CoV-RBD121-NP 45 µg + CpG. Vaccine preparations were stored at 4 °C for 1–2 months prior to administration.

Vaccinated animals were bled through the tail vein prior to immunization (pre-immune sera), at 12 days after the first vaccination, and at 14 days after the second vaccination. Terminal bleed sera were collected on day 42 (28 days post-vaccine dose 2) by terminal exsanguination after CO_2_ asphyxiation. Following terminal sera harvesting, spleens from two mice in each group were harvested for IFNγ ELISpot analysis.

All animal work performed in this manuscript has been conducted strictly in accordance with the “Guide for the care and use of laboratory animals” (8th edition), and other regulations established by the Office of Laboratory Animal Welfare (assurance number A4510-01). Because vaccine immune responses cannot be predicted, the simplest immunocompetent animal model for testing was used. Female inbred mice (BALB/c strain) were vaccinated, using the smallest number of animals that can give statistically significant measurements, typically five mice per group. All procedures were in accordance with ARRIVE guidelines (Touro University California IACUC identification code: TUCA006AM01M-01-2022; date of approval 18 January 2019).

### 2.6. IgG Detection by ELISA

IgG titers against CoV-RBD121 were measured by ELISA using S1-His (S1-His; 40591-V08H, Sino Biological, Beijing, China) to confirm recognition of the RBD portion. Briefly, 96-well plates (Nunc Maxisorb, ThermoFisher Scientific) were coated with 2.8 µg/mL of S1-His in 50 µL carbonate buffer (50 mM NaR_2_RCoR_3_R; pH 9.6) overnight at 4 °C. To calculate an absolute value of IgG as a standard, unlabeled mouse IgG (Southern Biotech, Birmingham, AL, USA) was coated at a starting value of 50 ng total IgG and then serially diluted 1:2 in carbonate buffer. Plates were washed (150 mM NaCl, 0.05% Triton X-100) and then blocked [2% bovine serum albumin (BSA) in 100 mM Tris pH 7.5, 0.5% Tween 20] for one hour at room temperature prior to incubation with 50 µL of a starting dilution of serum in PBS plus 2% BSA. For pre-immune sera, the starting dilution was 1:10, due to low expected titers. For bleed 1 sera post vaccine dose 1, starting dilution was also 1:10. For bleed 2 sera post vaccine dose 2, starting dilution was 1:100. Terminal bleed sera were evaluated at a 1:100 (for PBS or CoV-RBD121 45 µg groups) or at a 1:1000 starting dilution (all other groups). After a one-hour incubation at room temperature, plates were washed and incubated with 1:5000 dilution of goat anti-mouse IgG-HRP (Southern Biotech, Birmingham, AL, USA) in PBS + BSA. After one hour, plates were washed and then developed by a 3- to 5-min incubation with 50 µL TMB (Surmodics, Inc., Eden Prairie, MN, USA) and stopped with 50 µL 0.5 N HCl. Absorbance was read at 450 nm (Molecular Devices, San Jose, CA, USA), and the raw data were compiled by SoftMax Pro (Molecular Devices). Absolute values of IgG or isotype were determined by comparison of IgG mass against the standard, normalized by volume of sera in the assay. Graphs and statistical analysis between groups were completed using GraphPad Prism 8.0.2.

### 2.7. IFNγ Detection by ELISpot

First, 96-well plates (Millipore, Bedford, MA, USA) were coated with mouse antibodies recognizing IFNγ (BD Biosciences, San Jose, CA, USA) overnight at 4 °C in sterile water, and then washed and blocked according to the antibody manufacturer’s instructions. Single-cell suspensions of 2 × 10^5^ spleen cells from two animals in each group were plated in 200 μL RPMI + 10% FCS with or without 20 µg/mL S1-HIS protein (Sino Biological) or the irrelevant peptide DAPIYTNV as a negative control. Phorbol-12-myristate-13-acetate (PMA)/Ionomycin (2.5/250 ng/mL; Sigma, St. Louis, MO, USA) was used as a positive control. Cells were incubated for 36 h at 37 °C in a 5% CO_2_ incubator. At the incubation endpoint, cells were decanted, and plates were washed as per the manufacturer’s recommendations in water. IFNγ was detected with a biotinylated antibody against mouse IFNγ in PBS + 10% fetal bovine serum (FBS), followed by incubation with an Avidin-peroxidase (HRP) conjugate in PBS + 10% FBS. Plates were developed for HRP reactivity using AER substrate, according to the antibody manufacturer’s instructions (BD Biosciences, San Jose, CA, USA). After development, plates were washed in water and dried. IFNγ-positive spots (Intensity: 5–255; Size: 5–600; Gradient: 1–90) were counted on an EliSpot reader (AID, Strasbourg, Germany) and normalized to 10^6^ cells after background subtraction. Background was determined with stimulation with the peptide DAPIYTNV, typically less than 10 cells per million spleen cells.

### 2.8. VaxArray Coronavirus SeroAssay

The VaxArray Coronavirus SeroAssay Kit (#VXCV-5100, InDevR, Inc., Boulder, CO, USA) contains four microarray slides, printed with 16 replicate arrays per slide, PBB an optimized Protein Blocking Buffer (PBB, VX-6305), Wash Buffer 1 concentrate (VX-6303), and Wash Buffer 2 concentrate (VX-6304) Kit was used according to the manufacturer’s instructions as briefly described here. Prior to use, microarray slides were equilibrated to room temperature for 30 min in the provided foil pouch. Prepared standards and specimens were diluted at 1:100 in PBB and applied to the microarray, and microarray was incubated in a humidity chamber (VX-6200) on an orbital shaker at 80 rpm for 60 min. After incubation, samples were removed, and the microarray was washed with 50 μL of prepared Wash Buffer 1. Slides were washed for five minutes on an orbital shaker at 80 rpm after which the wash solution was removed with an 8-channel pipette. Anti-mouse IgG Label (VXCV-7620) was diluted 1:10 in PBB, aliquoted into 8-tube PCR strips, and 50 μL of label mixture was added to each microarray. Microarrays were incubated in the label mixture in the humidity chamber for 30 min, then sequentially washed in Wash Buffer 1, Wash Buffer 2, 70% ethanol, and ultrapure water. Slides were dried using a compressed air pump system and imaged using the VaxArray Imaging System (VX-6000).

### 2.9. SARS-CoV-2 Neutralization Assays

To calculate neutralization titers by PRNT50, sera from mice in the specified groups was collected on day 42, 21 days after they received two doses of PBS, antigen, or vaccine. Sera from each group were pooled and the dilution of serum that reduced 50% of plaques in a plate-based infection assay with the SARS-CoV-2 strain Wuhan-Hu-1 (Genbank ID: NC_045512) was determined with Vero E6 cells (JCRB no. JCRB1819). We incubated SARS-CoV-2 [100 plaque-forming unit (PFU)] with two-fold serial dilutions of pooled serum samples for one hour. The serum/virus mixture was then used to inoculate Vero E6 cells for 60 minutes. Cells were overlaid with EMEM agar medium plus 1.25% Avicel (DuPont, New Century, KS, USA), incubated for two days, and plaques were counted after staining with 1% crystal violet in formalin.

To calculate neutralization titers by the reduction in CPE, dilutions of heat-inactivated serum samples were mixed with 100× TCID50 (median tissue culture infectious dose) of the SARS-CoV-2 strain Wuhan-Hu-1 (Genbank ID: NC_045512) in duplicate and incubated for one hour. The serum/virus mixture was then added to Vero E6 cells (JCRB no. JCRB1819), and cells were incubated for three days at 37 °C and 5% CO_2_. Following incubation, the cell monolayers were fixed with 4% paraformaldehyde and stained with 1% crystal violet for 20 min at room temperature. Cells were then washed twice with 200 μL of filtered tap water and assessed visually for virus-induced CPE. The virus neutralization titer for each sample was reported as the reciprocal of the highest dilution that prevented CPE in 50% of the wells. Data are presented for each animal and a geometric mean titer (GMT) for the entire group was calculated.

To calculate neutralizing titers from the serum dilution that caused a 50% inhibition of infection of engineered 293T (BEI Resources–NR-52511) cells by S-pseudotyped lentivirus (pseudovirus, IC_50_), we used reagents and methods described in Crawford et al. (2020) [25]. The S used for this assay was from SARS-CoV-2 strain Wuhan-Hu-1 (Genbank NC_045512).

### 2.10. Statistical Analysis

Differences in antibody titers and neutralization activity were evaluated by one-way ANOVA with Tukey’s multiple comparisons. The correlation between neutralizing antibody titer and VaxArray signal for SARS-CoV-2 S protein was assessed by linear regression and the associated R^2^ value.

## 3. Results

### 3.1. Vaccine Design, Construction, and Stability

**Figure 1:**
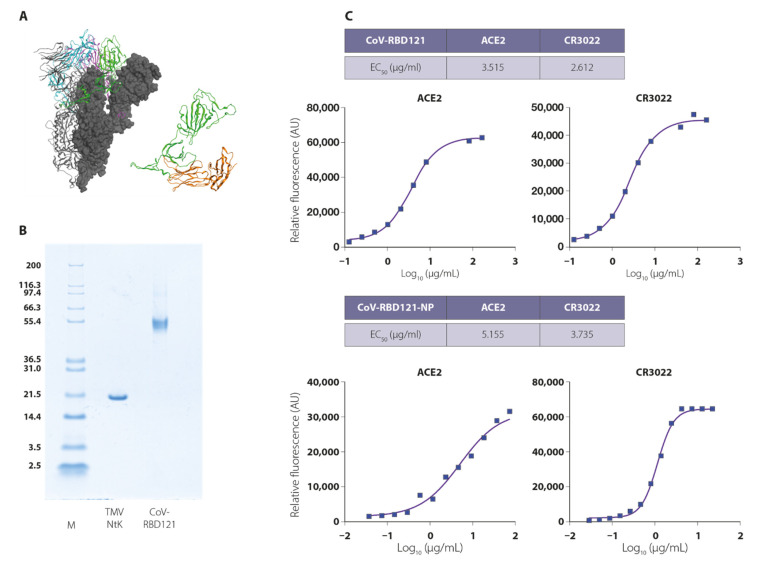
Potency of the CoV-RBD121-NP for ACE2 and the antibody CR3022. (**A**) Structural basis of the antigen based on S of SARS-CoV-2. (Left) SARS-CoV-2 S trimer is shown in ribbon and surface representation (PDB ID:6X6P). RBDs are colored green, magenta, and blue. (Right) Structure of the CoV-RBD121 antigen modeled with Molecular Operating Environment (MOE) software. The RBD (green) is amino acids 331–632 of SARS-CoV-2 S protein (PDB: 6X6P) and the Fc portion (orange) is from human IgG (PDB ID: 1HZH). (**B**) Purity of the TMV NtK virion (18.1 kDa) and of the CoV-RDB121 antigen (59.4 kDa) prior to conjugation as assessed by Coomassie staining of proteins separated by reducing SDS-PAGE. M indicates molecular markers. (**C**) Conformation and potency of the CoV-RBD121 antigen and the conjugated vaccine NP as assessed by binding to ACE2 and the monoclonal antibody CR3022.

Due to the association of full-length S protein from SARS1 and MERS with enhancement of viral infection in several preclinical systems [26,27,28,29] and serious pulmonary immune pathology in experimental animals [29,30,31,32,33], we designed an RBD vaccine antigen comprising amino acids 331–632 of the S1 component of the SARS-CoV-2 glycoprotein S (Figure 1A) [24]. To enhance antigen stability and facilitate at-scale purification, we fused the RBD to the constant domain (Fc) of a human IgG1 (allotype 171) [34,35,36]. The resulting antigen, CoV-RBD121 with a predicted molecular weight of 59.4 kDa, was produced in transfected *Nicotiana benthamiana* plants and purified to homogeneity (Figure 1B).

To confirm the conformation of the RBD, VaxArray analysis was performed to test for binding to the host virus receptor ACE2 and to the antibody CR3022. CR3022 recognizes a conserved epitope on the RBD and has neutralizing activity [37]. CoV-RBD121 bound to both ACE2 and CR3022 with an EC_50_ in the low micromolar range, indicating proper folding of the purified protein and folding of the RBD in a conformation that can induce neutralizing antibodies (Figure 1C, top).

A recombinant TMV strain with an N-terminal lysine residue (TMV NtK) [18] was produced in transfected *Nicotiana benthamiana* plants and purified to homogeneity (Figure 1B). CoV-RBD121 was conjugated to TMV NtK to produce the CoV-RBD121-NP vaccine formulation. The NP vaccine was formulated in a phosphate-buffered solution with 0.01% thimerosal as a preservative. Likewise, ACE2 and CR3022 VaxArray analysis confirmed that the RBD antigen was properly folded and displayed on the TMV virion (Figure 1C, bottom). The conjugate vaccine comprised of RBD domain chemically attached to TMV virion showed a similar ECR_50_R to the free RBD antigen, CoV-RBD121, both in the low micromolar range, for both ACE2 receptor and CR3022, RBD-binding monoclonal antibody.

**Figure 2:**
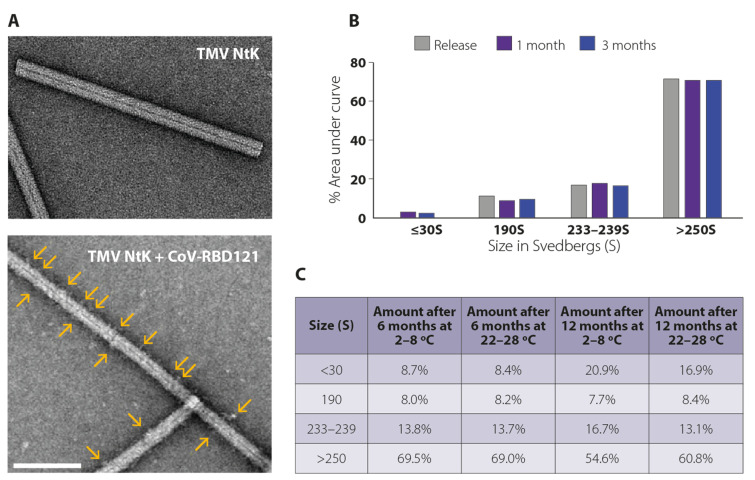
Physical properties of CoV-RBD121-NP. (**A**) Transmission electron microscopy of TMV Ntk virions and CoV-RBD121-NP (the virions after conjugation with the antigen). Yellow arrows indicate positions of conjugated antigen). Scale bar = 200 nm. (**B**) Size distribution of CoV-RBD121-NP at release and 1 and 3 months after storage at 2–8 °C. Distribution was calculated based on area under the curve (AUC) after analytical centrifugation. (**C**) Size distribution of CoV-RBD121-NP at 6 or 12 months after storage at 2–8 °C or 22–28 °C. Amount is shown as percentage of total based AUC analysis after ultracentrifugation.

The physical properties of the conjugate vaccine were characterized using several methods. Transmission electron microscopy (TEM) showed that the TMV NtK rods were uniform in shape with a diameter of <20 nm and an average length of 300 nm (Figure 2A, upper). The dense inner core structure, which contains the helically encapsidated TMV viral RNA, was observable throughout the length of the virion. Following conjugation, rods appeared fuzzy with less distinct edges, and the central dense core was partially obscured, indicating attachment of CoV-RBD121 (Figure 2A, lower). Conjugates were clearly observed as a raised-surface addition to TMV NtK (Figure 2A, yellow arrows).

We further characterized the NP vaccine using analytical ultracentrifugation. TMV NtK had a tight, two-peak sedimentation pattern with dominant peaks of 190–191*S* and 233–239*S* (Figure S1, top). Rarely, nonconjugated antigen monomer and dimer products were noted primarily in the 7–10*S* range. Compared with TMV NtK, CoV-RBD121-NP had a consistent shift in sedimentation to a broad sedimentation peak >250*S* (Figure S1, bottom). By sedimentation analysis, >95% of free antigen and >70% of TMV NtK were conjugated in the final product (Figure 2B).

**Figure 3:**
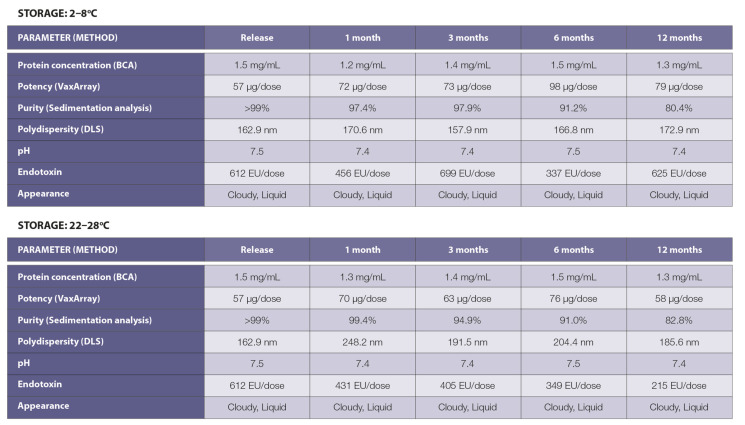
Properties of CoV-RBD121-NP after storage at 2–8 °C or 22–28 °C for up to 12 months. Protein concentration was assessed by bicinchoninic acid assay (BCA), potency by in vitro binding using a custom VaxArray for KBP, purity by AUC calculated from size distribution based on ultracentrifugation analysis, and polydispersity by dynamic light scattering (DLS).

Based on sedimentation analysis, the conjugated product was stable over a 12-month period at 2–8 °C or at 22–28 °C (Figure 2C). The conjugate vaccine appeared as a non-sedimenting cloudy solution due to the high concentration of TMV NtK particles. The appearance, pH, and protein concentration under both storage temperatures were stable throughout the 12-month period (Figure 3). Within the variation of the assay, no loss of vaccine potency occurred as measured by binding to antibody recognizing S. The polydispersity of the CoV-RBD121-NP was compared with TMV NtK using dynamic light scattering (DLS) methodologies. TMV NtK had an average DLS radius of 52.8 nm, the average DLS radius increased to 162.9 nm for the conjugate vaccine. The percentage of conjugate present as measured by sedimentation analysis (Figure 2C, Figure 3) was also consistent, suggesting little change in structural properties of the vaccine. The only noticeable difference after 12 months at either temperature was an increase in molecules in the <30*S* range that corresponded with a decrease in the molecules >250*S*, suggesting some dissociation of the vaccine (Figure 2C).

### 3.2. Vaccine Immunogenicity 

The ability of CoV-RBD121-NP to promote an immune response targeting SARS-CoV-2 was assessed in four vaccination tests in female C57BL/6 mice. On days 1 and 14, mice were immunized subcutaneously (s.c.) with CoV-RBD121-NP at 15 µg or 45 µg, with or without 7909 CpG adjuvant. These vaccinated mice were compared to mice injected with two doses of CoV-RBD121 (the antigen not conjugated to TMV) at 45 µg or to animals injected twice with vehicle (PBS + thimerosal). Blood was collected for antibody analysis on days 0, 12, and 28. On study day 42, mice were euthanized, terminal sera were collected, and spleens were harvested for IFNγ analysis.

**Figure 4:**
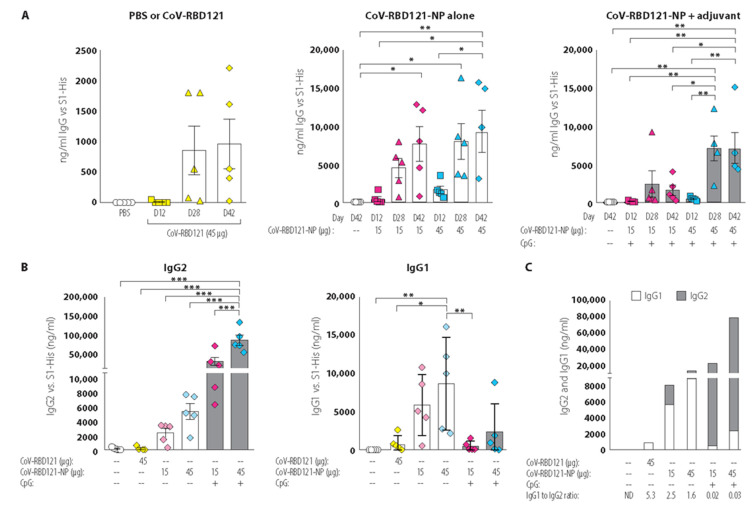
Immune response to CoV-RBD121 or CoV-RBD121-NP with or without adjuvant. (**A**) Concentration of IgG recognizing S1-His in sera from mice administered the indicated antigen or vaccines. CoV-RBD121 is the antigen alone, CoVRBD121-NP is the TMV-conjugated NP vaccine. Sera were collected on day 12 (D12) after the first dose, and on day 28 (D28), 14 days after the second injection dose. Terminal sera were collected on day 42 (D42), 28 days after boosting. For mice receiving PBS, data for sera collected on day 42 are shown. (**B**) Concentration of IgG2 (left) and IgG1 (right) recognizing S1-His in sera collected from mice on day 42 administered the indicated antigen or vaccines. (**C**) Stacked bar graph shows the amount of IgG1 and IgG2 antibodies recognizing S1-His, which were used to calculate the ratio of IgG1 to IgG2. Significant differences among the groups were determined by one-way ANOVA with Tukey’s correction for multiple comparisons (*n* = 4–5 mice per group as indicated; * *p* ≤ 0.05; ** *p* ≤ 0.01; *** *p* ≤ 0.001). Colors are coordinated to indicate individual values for mice immunized with PBS (clear); CoV-RBD121 antigen alone (yellow); 15 mcg CoV-RBD121-NP (magenta); and 45 mcg CoV-RBD121-NP (blue) at day zero (circles), Day 12 post vaccination (squares), Day 28 post vaccination (triangles) and Day 42 post vaccination (diamonds).

The collected serum was tested for total IgG reactivity against RBD by ELISA, using recombinant histidine-tagged S1 (S1-His) from SARS-CoV-2 as the capture antigen (Figure 4). Some mice had a low response to immunization with the unconjugated antigen CoV-RBD121 on day 28 and day 42 (Figure 4A, left). Most of the mice receiving the 15 µg dose of CoV-RBD121-NP with or without adjuvant exhibited a low IgG response after the first immunization (day 12) (Figure 4, middle and right). In the non-adjuvanted formulation, a strong boosting effect was observed on day 28 in mice receiving either the 15 µg or 45 µg dose (Figure 4A, middle). With adjuvanted formulation, the mice receiving the 45 µg dose produced a strong IgG response by day 28 that was similar at day 42 (Figure 4A, right). The limited response to the unconjugated antigen CoV-RBD121 compared to that with the TMV-conjugated vaccine CoV-RBD121-NP indicated that conjugation to the TMV NtK NP is needed for a strong immune response.

Given the importance of the balance between the two types of helper T cells, Th1 and Th2 cells, in an effective immune response to many pathogens, we performed IgG isotype analysis. We measured IgG1, as an indication of a Th2 response, and IgG2, both IgG2a and IgG2c, as an indication of a Th1 response, on sera from day 42 (Figure 4B). As expected, the PBS (control) and CoV-RBD121 groups had undetectable or low concentrations of either IgG1 or IgG2 antibodies. Not only did IgG2 titers increase with increasing dose, but these antibodies were also markedly induced in the mice that received CoV-RBD121-NP at either dose with the CpG adjuvant (Figure 4B, left). In contrast, IgG1 titers were significantly higher in mice receiving the non-adjuvanted vaccine (Figure 4B, right). These differences were reflected in the ratio of IgG1 to IgG2 isotype, with an IgG1 to G2 ratio greater than 1 for animals receiving non-adjuvanted CoV-RBD121-NP and an IgG1 to G2 ratio less than 0.1 for animals receiving CoV-RBD121-NP with CpG adjuvant (Figure 4C), indicating a class switch was induced by the CpG adjuvant.

Consistent with the stronger Th1 cell response in response to CoV-RBD121-NP with adjuvant indicated by the IgG isotype analysis, we observed higher numbers of IFNγ-secreting cells in cells isolated from the spleens of mice receiving the adjuvanted formulation (Figure S2). In contrast, cells from mice injected with CoV-RBD121 or CoV-RDB121-NP without adjuvant did not have any more IFNγ-producing cells than those isolated from vehicle-treated mice.

### 3.3. Vaccine-Induced Neutralizing Antibody Titers

**Figure 5:**
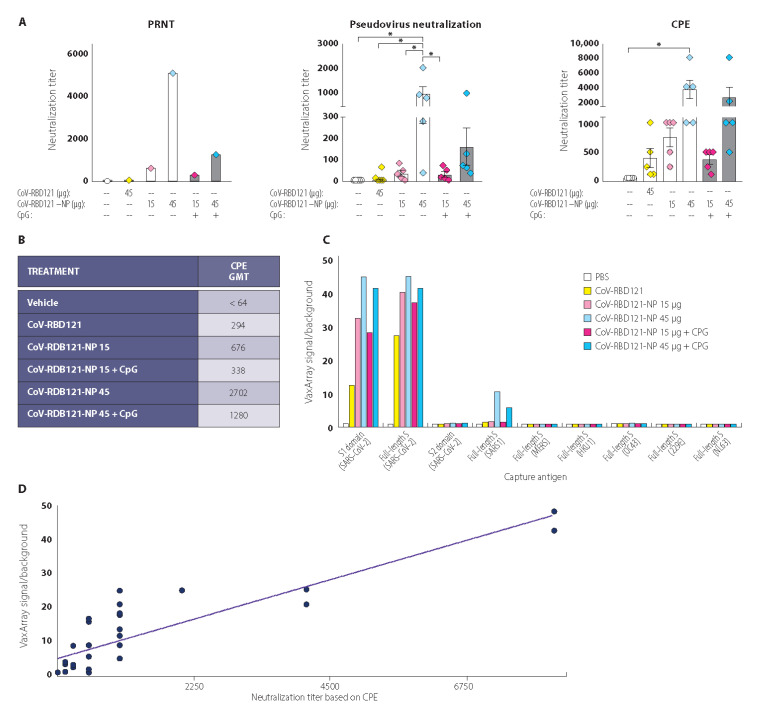
Induction of neutralizing antibodies and antibody specificity by CoV-RBD121 or CoV-RBD121-NP. (**A**) Neutralizing activity of the antibodies in the mice was evaluated in sera collected on day 42. For the PRNT assay, sera were pooled because quantities were limiting. For the pseudovirus neutralization assay or CPE assay, results for each mouse are shown. Significant differences among the groups were determined by one-way ANOVA with Tukey’s correction for multiple comparisons (*n* = 4–5 mice per group; * *p* ≤ 0.05). (**B**) CPE data were used to calculate a GMT for each group. (**C**) Specificity of the antibody response was evaluated by VaxArray SeroAssay. Results from day 42 pooled sera. Data are presented as the ratio of signal to background. (**D**) Correlation analysis for neutralization titer for each mouse based on CPE results and VaxArray results for S1. Results of linear regression analysis and R^2^ are provided. Significant differences among the groups were determined by one-way ANOVA with Tukey’s correction for multiple comparisons (*n* = 4–5 mice per group as indicated; * *p* ≤ 0.05). Colors are coordinated to indicate individual values for mice immunized with PBS (clear); CoV-RBD121 antigen alone (yellow); 15 mcg CoV-RBD121-NP (magenta); and 45 mcg CoV-RBD121-NP (blue) at day zero (circles), Day 12 post vaccination (squares), Day 28 post vaccination (triangles) and Day 42 post vaccination (diamonds).

Although IgG titer, IgG isotype ratios, and cellular immunity are important measures of vaccine performance, a critical measure of immunity is the ability of the stimulated immune response to neutralize viral infection by blocking cellular entry. We performed three different in vitro assays for neutralization activity of the antibodies (Figure 5A). Because the amount of serum collected from each mouse was limited, for two of the assays samples from each mouse were evaluated individually. For the PRNT assay, sera from the mice for the individual treatments were pooled. By identifying the dilution of pooled sera that reduced 50% of plaques of SARS-CoV-2 virus in VeroE6 cells (PRNT50), we found that non-adjuvanted CoV-RBD121-NP at the 45 µg dose produced the highest value (Figure 5A, left). The highest neutralizing titers were also obtained with non-adjuvanted CoV-RBD121-NP at the 45 µg dose in an assay based on the dilution that produced a reduction in infection of cells with a pseudovirus coated with SARS-CoV-2 S protein (Figure 5A, middle). In a neutralization assay based on the serum dilution that produced a 50% reduction in the cytopathic effect (CPE) after infection with SARS-CoV-2 in VeroE6 cells, we detected high titers in animals receiving either of the 45 µg doses of CoV-RBD121-NP with or without adjuvant (Figure 5A, right). Using the CPE data, we calculated a geometric mean titer (GMT) for each treatment group (Figure 5B). These results indicated a clear dose response to the candidate vaccine and no benefit from the adjuvant CpG in stimulating neutralization titers, despite clear differences in the IgG1/IgG2 ratio (Figure 4C).

### 3.4. Reactivity with Diverse Coronavirus Spike Proteins

Using a VaxArray for coronaviruses, we tested pooled day 42 sera for antibodies that reacted with S proteins from seven coronaviruses, as well as for reactivity with the S1 and S2 domains from SARS-CoV-2 (Figure 5C). Serum from mice in any of the four CoV-RBD121-NP groups or the group that received antigen alone (CoV-RBD121) contained antibodies that reacted with the full-length S protein of SARS-CoV-2. Mice in the four CoV-RBD121-NP groups also had strong reactivity toward the S1 domain and a comparatively low amount of reactivity to S1 was detected with sera from the mice in the CoV-RBD121 group. No reactivity was noted with to SARS-CoV-2 S2 domain or with S proteins the other coronaviruses tested. A weak signal was detected against SARS S protein in animals receiving either adjuvanted or non-adjuvanted CoV-RBD121-NP at the 45 µg dose.

To determine if virus neutralization titers corresponded to antibody reactivity against the SARS-CoV-2 S protein, we determined the correlation between the VaxArray data for the mice in the CoV-RBD121 group or any of the CoV-RBD121-NP groups and the neutralization titers based on CPE analysis for each mouse in these groups (Figure 5D). This analysis revealed a good correlation (R^2^ 0.77) between neutralizing titers and the VaxArray signal for the S1 domain.

## 4. Discussion

The COVID-19 pandemic has spurred the development of vaccine candidates utilizing a wide range of vaccine platforms [5]. Various vaccines based on inactivated SARS-CoV-2, nucleic acid, replication-competent or incompetent virus vectors, as well as recombinant spike proteins, including those presented as VLPs, are under clinical development. Further, NP vaccine platforms are emerging as promising systems [38,39]. A plant-based VLP vaccine coupled with the 1018 CpG or AS02 adjuvant induced high neutralizing antibody titers in human patients [38]. These data, along with the attractive safety data, show the strong potential for NP vaccines to induce the type and strength of immune response needed in a SARS-CoV-2 vaccine. Key to the success of a vaccine platform for worldwide use is manufacturing consistency and stability at low cost and with widely available systems. Extreme storage conditions (–20 to –80 °C), such as those required for the mRNA-based vaccines by Pfizer/BioNTech and Moderna, create logistical barriers to worldwide use [40]. Vaccine systems that induce strong immunological responses and are stable under standard refrigeration or even at room temperature are urgently needed to address this global pandemic.

Here, we report a robust and rapid platform for recombinant antigen expression in *N. benthamiana*—purification and chemical conjugation to TMV NtK virions for the presentation of an RBD-based antigen of the S protein of SARS-CoV-2. The vaccine preparations exhibited remarkable consistency and stability for up to 12 months at 2–8 °C or even 22–28 °C, making this formulation practical for global distribution. Compared with other studies of NP-based SARS-CoV-2 vaccine platforms [38,40,41], we provided multiple lines of evidence demonstrating stable physical and biochemical properties of the nanoparticle vaccine (CoV-RBD121-NP) across 12 months at two logistically practical temperatures. Through conjugation of the SARS-CoV-2 antigen to the TMV particle, our platform conferred VLP features to a soluble antigen, thereby augmenting immune stimulation compared with that achieved with the antigen alone.

The vaccine contains inactivated (through UV inactivation) TMV viral RNA. Other vaccines with a similar TMV NP have been tested in clinical trials with children for malaria (ClinicalTrials.gov, accessed on 4 October 2021, Identifiers: NCT00380393A; NCT02992119). The results from one of these trials suggests that the TMV NP does not represent a safety concern [42,43]. Additionally, the RNA in VLPs stimulates the receptor TLR7 and this signal is required for the differentiation of memory B cells, formation of secondary plasma cells, and high-affinity antibody production [44]. Thus, this inactivated viral RNA in the TMV NP formulation may be beneficial by stimulating the immune response.

We selected the RBD as the antigen because this region of the S protein is the dominant target for neutralizing response in COVID-19 infection [37,45,46,47] and because the full-length S protein of other coronaviruses was associated with enhancement of viral infection or pulmonary toxicity in other studies [26,27,29,30,31,32,33]. Consistent with a previous study [9,10,11], we found that the RBD alone was a weak immunogen and that coupling the antigenic fusion protein to the TMV virion resulted in a >10-fold enhancement in overall IgG titer compared with that achieved with CoV-RBD121 alone. The antigen is a fusion protein between RBD (amino acids 331–632) and human Fc. Consequently, the mice showed strong reactions to the human Fc component, as well as to RBD. We expect that this Fc component would not be immunogenic in humans.

Although the TMV particle itself is foreign to humans and is a potent immunogen [9,10,11], we tested formulations with the adjuvant CpG 7909 and without this adjuvant. Interestingly, both formulations stimulated strong immune responses, however, CoV-RBD121-NP with CpG 7909 biased the isotype response toward a Th1 type, and CoV-RBD121-NP without the adjuvant resulted in more balanced Th1 and Th2 responses. In contrast to CoV-RBD121-NP, the unconjugated free antigen stimulated primarily a Th2 response. Ideal COVID-19 vaccination strategies induce a combination of antibody and cellular immune responses [48]. We found that the addition of CpG 7909 resulted in the induction of cells that secreted IFNγ in the spleens of immunized animals. The induction of such cells indicated the expansion of T cells among other immune cells [49,50]. Thus, the induction of IgG antibodies along with the induction of IFNγ-secreting cells suggested that this formulation could produce an advantageous combination of antibody and cellular immune responses.

Although in vitro assays to detect the presence of neutralizing antibodies cannot completely predict in vivo protection afforded by a vaccine, such assays are useful for screening vaccine candidates. Because each assay has limitations, we used three different assays of neutralizing antibody titer to evaluate the effectiveness of the CoV-RBD121-NP in both adjuvanted and non-adjuvanted formulations. Not only did the use of three assays provide strong support for the production of neutralizing antibodies, but we found that the CPE assay was the most sensitive in detecting low concentrations of such antibodies. Furthermore, we found that all three assays provided qualitatively similar results, showing the strongest neutralizing antibody induction with the 45 µg dose of CoV-RBD121-NP with or without adjuvant.

Consistent with the immunogenic properties of the VLP-based NPs [8], even association of the RBD antigen with TMV NtK greatly increased neutralizing antibody titers induced in mice. These data are also consistent with the results of other RBD NP vaccines [39]. In the neutralizing antibody assays, the non-adjuvant formulation was better at stimulating neutralizing antibodies. The overall effect of CpG 7909 adjuvant was complex, not only affecting the production of neutralizing antibodies but also the balance of the response in terms of Th1 or Th2. However, both adjuvanted and non-adjuvanted formulations had neutralizing activity based on the three in vitro assays.

We received the sequence of the antigen and designed and manufactured the pilot batch of this TMV-based NP vaccine within 28 days with only 14 days needed from the time the RBD-encoding plasmids were ready for purification of the first batch of vaccine candidates. With the data presented here, we demonstrated that the TMV-based NP vaccine platform can be readily adapted to a new vaccine target, such as SARS-CoV-2 or other emergent variant(s), enabling the formulation of consistent and stable conjugate vaccine products. Furthermore, the vaccine candidate CoV-RBD121-NP produced antigen-relevant immune responses with high neutralizing antibody titers. Collectively, the data indicate that such a vaccine has the potential to protect against SARS-CoV-2 and should continue undergoing evaluation as a COVID-19 vaccine candidate.

## Data Availability

The data is contained within the article or supplementary material.

## Supplementary Materials

The following are available online at https://www.mdpi.com/article/10.3390/vaccines9111347/s1, Figure S1: Sedimentation coefficient distribution for TMV NtK and CoV-RBD121-NP. Figure S2: Analysis of IFNχ-producing cells from spleens of mice receiving CoV-RBD121 or the unadjuvanted or adjuvanted formulations of CoV-RBD121-NP.
