# Supplementary material for: Development of a SARS-CoV-2 Vaccine Candidate Using Plant-Based Manufacturing and a Tobacco Mosaic Virus-Like Nano-Particle
Source: Vaccines (Basel). 2021 Nov 17;9(11):1347. doi: 10.3390/vaccines9111347 (PMC8619098; doi:10.3390/vaccines9111347)
Supplement: Supplementary file 1 [file vaccines-09-01347-s001.zip › vaccines-1377465-supplementary.pdf]

Development of a SARS-CoV-2 vaccine candidate using plant-based manufacturing and a tobacco mosaic virus-like nanoparticle

Supplementary Materials

figure S1. Sedimentation coefficient distribution for TMV NtK and CoV-RBD121-NP.

figure S2. Analysis of IFNχ-producing cells from spleens of mice receiving CoV-RBD121 or the unadjuvanted or adjuvanted formulations of CoV-RBD121-NP.

Supplementary figures

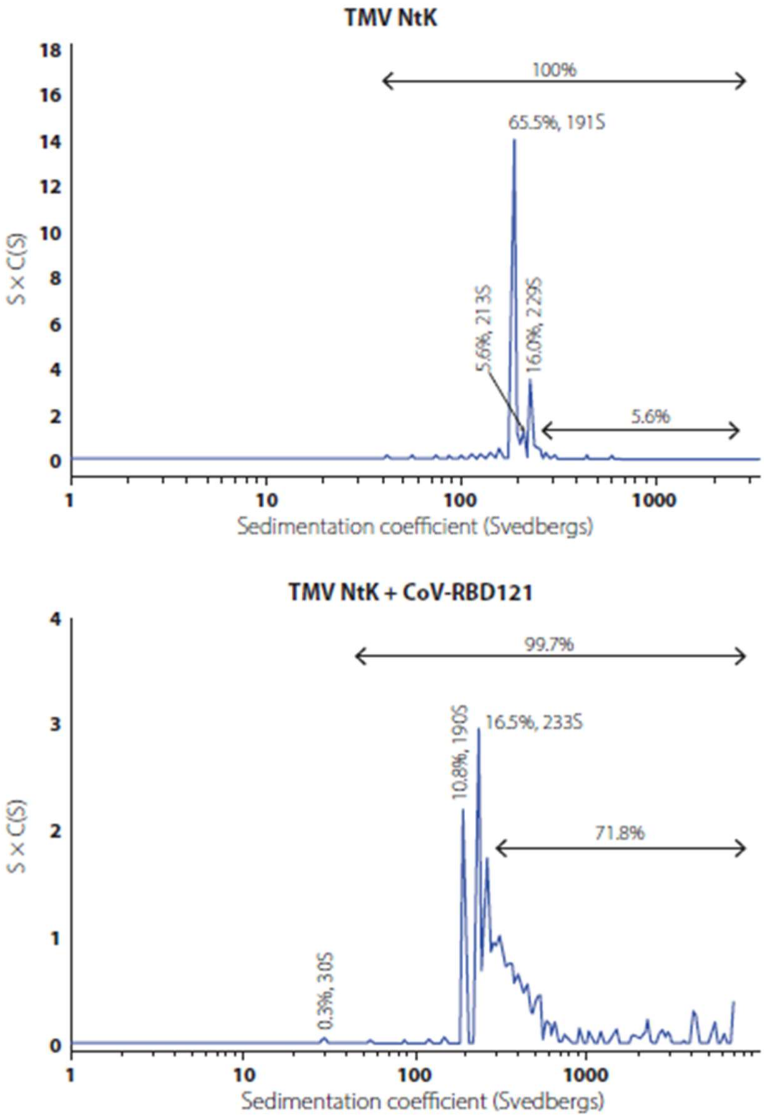

figure S1. Sedimentation coefficient distribution for TMV NtK and CoV-RBD121-NP. Data are from ultracentrifugation of a sample of TMV NtK before conjugation to the antigen and after conjugation to COV-RBD121.

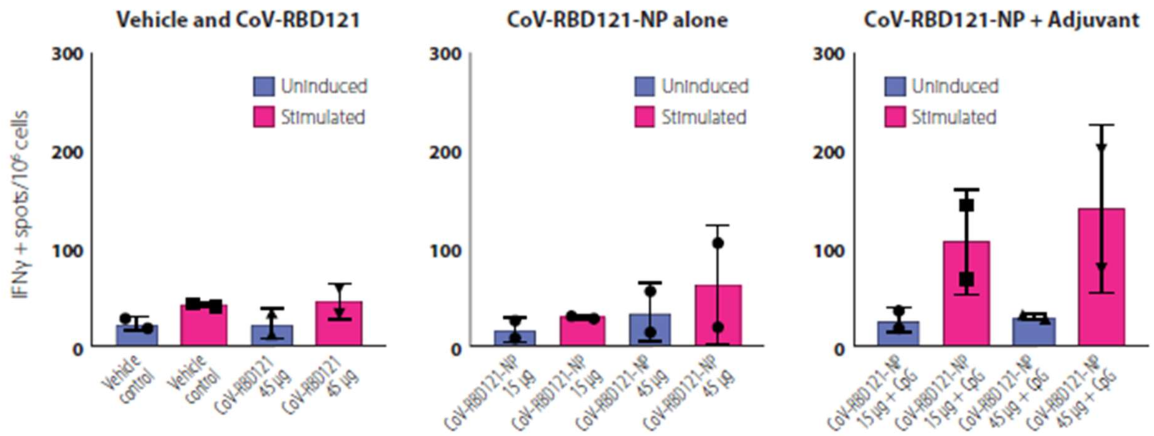

figure S2. Analysis of IFNχ-producing cells from spleens of mice receiving CoV-RBD121 or the unadjuvanted or adjuvanted formulations of CoV-RBD121-NP. Data show the results of ELISpot assay from the cells from the spleens of both mice harvested from each treatment group. Stimulated indicates that the cells were incubated with S1-HIS protein to detect a response to the RBD. Uninduced cells were not exposed to S1-His and represent the background number of IFNγ-positive cells.
